# Hypothesis for potential pathogenesis of SARS-CoV-2 infection–a review of immune changes in patients with viral pneumonia

**DOI:** 10.1080/22221751.2020.1746199

**Published:** 2020-03-30

**Authors:** Ling Lin, Lianfeng Lu, Wei Cao, Taisheng Li

**Affiliations:** a Department of Infectious Diseases, Peking Union Medical College Hospital, Peking Union Medical College, Chinese Academy of Medical Sciences, Beijing, People’s Republic of China; b Center for AIDS Research, Chinese Academy of Medical Sciences and Peking Union Medical College, Beijing, People’s Republic of China; c Clinical Immunology Center, Chinese Academy of Medical Sciences, Beijing, People’s Republic of China; d Tsinghua-Peking Center for Life Sciences, School of Medicine, Tsinghua University, Beijing, People’s Republic of China

**Keywords:** COVID-19, SARS-CoV-2, anticoagulation, IVIg, pathogenesis

## Abstract

Coronavirus disease 2019 (COVID-19) is an infectious disease caused by severe acute respiratory syndrome coronavirus 2 (SARS-CoV-2) with droplets and contact as the main means of transmission. Since the first case appeared in Wuhan, China, in December 2019, the outbreak has gradually spread nationwide. Up to now, according to official data released by the Chinese health commission, the number of newly diagnosed patients has been declining, and the epidemic is gradually being controlled. Although most patients have mild symptoms and good prognosis after infection, some patients developed severe and die from multiple organ complications. The pathogenesis of SARS-CoV-2 infection in humans remains unclear. Immune function is a strong defense against invasive pathogens and there is currently no specific antiviral drug against the virus. This article reviews the immunological changes of coronaviruses like SARS, MERS and other viral pneumonia similar to SARS-CoV-2. Combined with the published literature, the potential pathogenesis of COVID-19 is inferred, and the treatment recommendations for giving high-doses intravenous immunoglobulin and low-molecular-weight heparin anticoagulant therapy to severe type patients are proposed.

Coronaviruses (CoVs) have been on the top news again after the severe acute respiratory syndrome coronavirus (SARS-CoV) in 2003 [1] and Middle East Respiratory Syndrome(MERS-CoV) outbreak in Saudi Arabia and south korea [2], since a new Coronavirus occurred in Wuhan, Hubei province and quickly spread across over whole China and other 30 countries. Up to date (25 February 2020), there are approximately 80000 infectious patients confirmed and caused above 2500 death [3], which need urgent viral infection identification and intervention as early as possible. Although nucleic acid testing played an utmost part in the detection of the viral genome sequence [4], over-hypersensitivity and dependence on sampling may lead to some degree of false-positive or false-negative in the clinic. Despite several types of research have been conducted to practical treatments or any possible medicines, the consensus has not recommended any antiviral medicine [5]. It has been confirmed that the immune system played a vital role in defense against SARS-CoV and MERS infection. Immune changes in patients with SARS [6], MERS [7] and influenza [8], especially changes in peripheral blood T lymphocyte subsets, contribute to understanding the characteristics, diagnosis, monitoring, prevention and treatment of the disease.

## Immune changes in SARS

In the acute phase of SARS-CoV infection, rapid reduction of lymphocytes in peripheral blood [6], mainly T lymphocytes, was observed, and both CD4+ and CD8+ T lymphocytes were decreased. The loss of lymphocytes precedes even the abnormal changes on the chest X-ray [9,10]. After a one-year follow-up of SARS patients, CD3+, CD4+, and CD8+ T cells recovered rapidly during the disease recovery period. CD8+ T lymphocytes, which returned to normal within 2–3 months after onset. The memory CD4+ T cells returned to normal a year after onset, whereas other cell counts including total T lymphocytes, CD3+ cells, CD4+ cells, and naive CD4+ T cells were still lower than healthy controls [11]. It assumed that after a viral infection, lymphocytopenia in peripheral blood due to lymphocyte sequestration firstly. The increase in lymphocytes during recovery is not new cells produced by the thymus, but lymphocyte recirculation between peripheral blood and tissues or organs [11]. SARS-specific IgG antibodies are produced in the late acute stage about 2 weeks and gradually increase with the course of the disease [12]. The sustainable existence of IgG makes the patients acquire the immune function after infection. Recovering patients have high and sustained levels of S protein-specific neutralizing antibody responses, which may play an important role in determining disease outcome [13]. The IgG level of mild patients was significantly higher than that of severe patients. In the course of disease progression, patients may be accompanied by increased IL-8 and TNF-α levels, which peak in the early stage of recovery, while MCP-1 shows a rapid increase in the early acute stage and gradually decreases with the progress of the disease. Based on the above findings, it is proposed to treat the disease with a low-dose of glucocorticoid for less than 2 weeks in the early stage of the disease, so that the symptoms of most severe patients are well controlled [9]. This is different from the effect of high-dose of glucocorticoids described in other literature [14].

## Immune changes in MERS

A previously unknown coronavirus (MERS-CoV) which isolated from a patient in 2012 [15] and caused high mortality rates in family-based and hospital-based outbreaks [2], was similar to severe acute respiratory syndrome coronavirus (SARS-CoV). The genomic structures of the two viruses are very similar, both single-stranded positive-sense RNA viruses, but when they enter host cells, MERS-CoV and SARS-CoV attach to different receptor dipeptidyl peptidase 4(DDP4) and angiotensin-converting enzyme 2 (ACE2) respectively [16,17]. Besides, the clinical manifestations of MERS-CoV infection, like SARS-CoV, range from asymptomatic infection to severe pneumonia with acute respiratory distress syndrome, septic shock, and multi-organ failure resulting in death [15]. Though the clinical pathogenesis have not been explained clearly, a retrospective study [7] comparing 45 patients in South Korea has suggested that a decreasing number of peripheral lymphocyte (lymphopenia as an absolute lymphocyte count lower than 1,000 cells/mm3), thrombocytopenia (platelet count lower than 150000 cells/mm3) and high CRP level could predict pneumonia development and progression to respiratory failure at the early course of the disease. This result is supported by evidence of the report by Min et al. [18]. Whereas it should be noteworthy that lymphopenia in MERS infection is not significant as SARS patients [19], and research found that except immunosuppression state and concomitant infection, age was the only predictor [20].

Concerning MERS-CoV T cell responses, within two weeks after the onset of symptoms (acute phase), besides pro-inflammatory cytokine/chemokine secretion, such as interleukin (IL)-6 and C-X-C motif chemokine (CXCL)-8 [21,22], high frequencies of MERS-CoV-reactive CD8+ T cells were observed in patients with severe/moderate illness, which before the detection of humoral and CD4+ T cell responses. During the convalescent phase, the magnitude of the CD8+ T cell response was not greatly augmented [22]. This result indicates that the inefficient control of invading MERS-CoV brings about robust inflammatory and CTL responses, which play a vital role in clearing the virus. Further, PBMCs obtained on day 24 after illness onset shows a strong specific T-cell response against the MERS-CoV S protein [23]. Role of cytotoxic T lymphocytes (CTL) protection from MHV(a member of the same beta coronavirus group as SARS-CoV and MERS-CoV) virus clearance also been demonstrated in animal experimental models [24].

Zhao, J et al. [25] found that during the convalescent phase, neutralizing (PRNT50) antibody titers measured in vitro predicted serum protective ability in infected mice and correlated with CD4 but not CD8+ T cell responses. And in their experiment, the CD4+ T cells were phenotypically effector memory (CD45RA−CCR7−) cells, while the virus-specific CD8+ T cell populations were half effector (CD45RA-CCR7−) cells and half CD45RA+CCR7− cells. Persistent and gradual increases of lymphocyte responses after symptom onset in MERS patients may be required for effective immune responses against MERS-CoV, but whether the CTL turnover and what the effect these cells provide still need further research.

## Immune changes in influenza

Different from the disappearance of MERS and SARS, the influenza pandemic (H1N1) in 2009 been incorporated into seasonal strains. However, the influenza pandemic (H1N1) has a relatively high mortality rate [26] compared to influenza A virus (IAV) and influenza B virus (IBV) which are usually coved in seasonal strains [27]. The influenza genome contains 8 segments and is capable of both antigenic drift and shift, making small nucleotide mutations and the exchange of genome segments respectively. Due to these characteristics, novel antigens never existed in the human population to make seasonal epidemics possible.

The manifestation of H1N1 infections ranged from subclinical symptoms to significant malaise with fever, myalgias, and rhinorrhea, progressing to acute hypoxemia and acute respiratory distress. Extra-pulmonary presentations like gastrointestinal, neurologic and cardiac dysfunction, have also been described in the review [26]. Albeit pandemic 2009 H1N1 virus infection and host immunity patterns are incompletely characterized, in the early response period, increased plasma levels of IL-15, IL-8, and especially IL-6 may be markers of critical illness [28]. Another study [28] mapped 41 healthy volunteer T cell responses to influenza before and during infection and demonstrated that influenza-specific CD4+ T cells correlate with disease protection against influenza challenge in humans. However, Kristin G.I. Mohn et al. [8] stimulated PBMC during acute and convalescent patients, suggesting that significantly lower frequencies of influenza-specific CD8+ compared with CD4+ IFN-γ T-cells in acute patients, while high levels of both CD4+ and CD8+ T cells directed against conserved core antigens in convalescent patients. Considering the lack of enough sample size and study design, whether there is a discrepancy of T lymphocyte subgroups dominance in different periods of infection should be explored further, but the CD4+ T lymphocyte counts have been linked to disease severity [28,29].

It is noteworthy that in the seasonal influenza infection, peripheral lymphocyte counts and lymphocyte subsets showed a significant correlation with prognosis of severe illness. Studies compared IAV or IBV patients with healthy subjects, elevated serum IL-6, IL-8 level and decreased CD3+CD4+, CD3+CD8+, NK counts related to patients infection [30,31].

As reviewed in this literature, the viruses of respiratory infections like SARS-CoV, MERS-CoV and influenza virus all caused periodical outbreak and death. Histopathological examinations show common characteristics: diffuse alveolar damage, edematous lung lesions and pneumonia [32–34]. Besides, the examination of virus infection demonstrates that the capability of virus replication efficiently in the upper and lower respiratory tract is associated with mild or moderate clinical signs and pathological changes [33–35], though only in the MERS-CoV infected rhesus macaque could detect transient lymphocyte cell reduction in the first 2 days after inoculation [33]. Jiang Gu et al. [36] hypothesized that the marked T lymphocytes (CD3+, CD4+, and CD8+ cells) decline weakened immune system and aggravated the SARS infection of the respiratory tract. In summary, virus replication, distribution and associated immune response contributed to the progress of the infectious disease. The change of peripheral lymphocyte change and the transition of lymphocyte subgroups may provide new thoughts for the pathogenesis of SARS-CoV-2 infection.

## Hypothetical pathogenesis

Based on the published literature and clinical observations of COVID-19 patients, we propose reasonable hypotheses about the pathogenesis of SARS-CoV-2 infection in humans. The virus might pass through the mucous membranes, especially nasal and larynx mucosa, then enters the lungs through the respiratory tract. The early most common symptoms of infection are fever and cough [37]. The virus may enter the peripheral blood from the lungs, causing viremia. Then the virus would attack the targeting organs that express ACE2, such as the lungs, heart, renal, gastrointestinal tract [38,39]. The SARS-CoV-2 detected in the fecal samples [37] is more likely because the virus enters the blood from the lungs and then travels from the blood to the intestines, which supports our hypothesis. Dawei Wang et al found that the median time from symptom onset to ARDS was about 8 days [40]. We speculate that in this way, the virus begins a second attack, causing the patient's condition to aggravate around 7–14 days after onset. During the infection process, the white blood cell count in peripheral blood in the early stage of the disease is normal or slightly low [37], and lymphopenia is observed in patients [40]. We speculate that B lymphocyte reduction may occur early in the disease, which may affect antibody production in the patient. In severe type patients, lymphocytes were significantly reduced [40]. We speculate that lymphocytes in patients with COVID-19 might gradually decrease as the disease progress. But the mechanism of significant lymphocyte reduction in severe type patients remains unclear. Besides, the inflammatory factors associated with diseases mainly containing IL-6 [41] were significantly increased, which also contributed to the aggravation of the disease around 7–14 days after onset. Non-survivors had higher levels of neutrophils, D-Dimer, blood urea nitrogen, and creatinine than the survivors [40].

**Figure 1.**
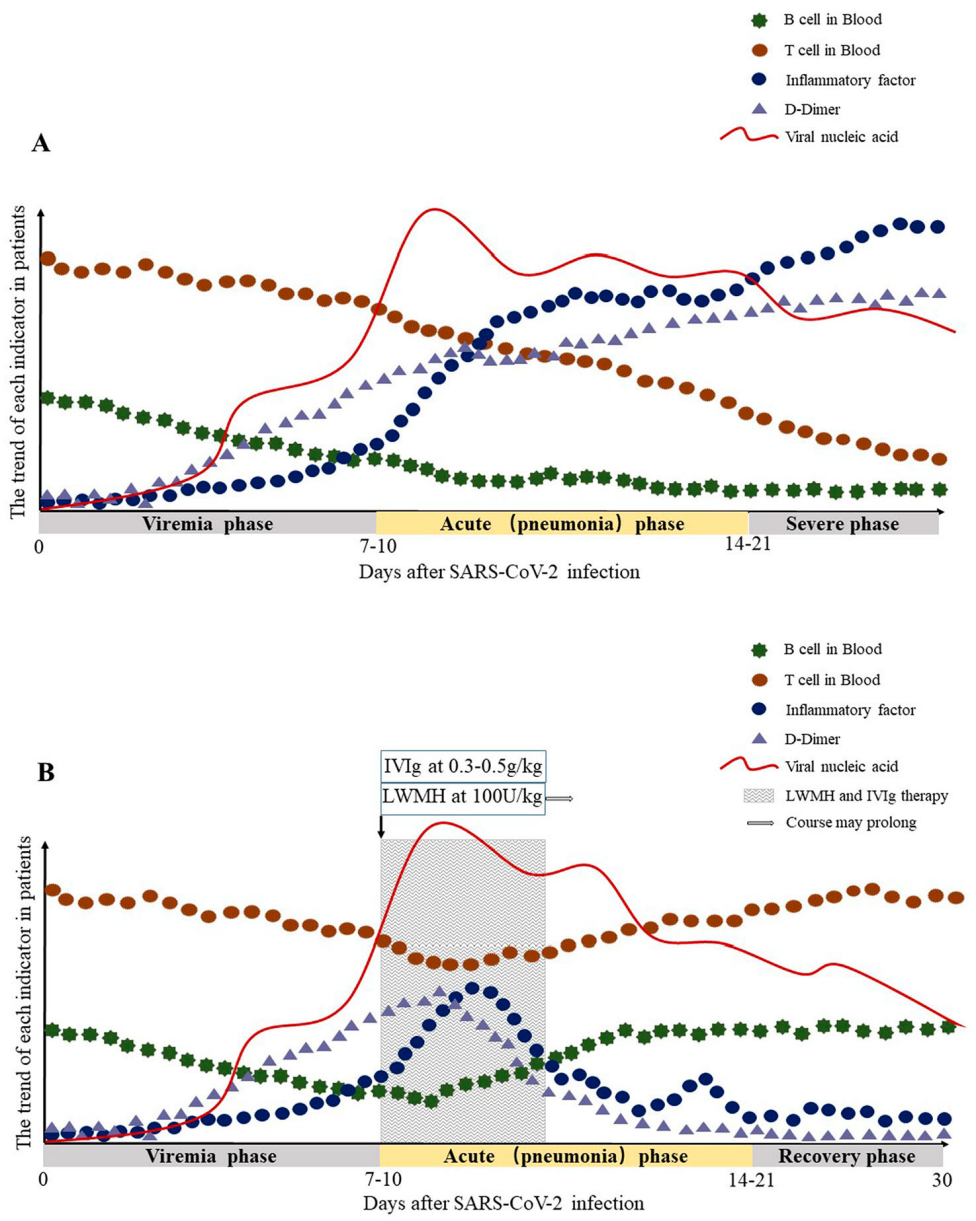
**Hypothetical pathogenesis of COVID-19**. The X-axis is the number of days after SARS-CoV-2 infection, and it is divided into three phases according to the above conjecture. The Y-axis is the trend of T cells, B cells, inflammatory factors, D-Dimer and viral load in patients. (A) The trend of each indicator in COVID-19 patients with severe type; (B) The trend of each indicator in COVID-19 patients with severe type after LWMH and IVIg therapy. The shaded areas represent the recommended intervention times for LMWH and IVIg treatment.

Based on the above assumptions, the clinical phase is divided into three: the viremia phase, the acute phase (pneumonia phase) and the recovery phase. If the immune function of patients in the acute phase (pneumonia phase) is effective, and no more basic diseases, the virus can be effectively suppressed, then enter the recovery phase. If the patient is older, or in an immune impaired state, combined with other basic diseases such as hypertension and diabetes, the immune system cannot effectively control the virus in the acute phase (pneumonia phase), the patient will become severe or critical type. As we mentioned in our hypothesis, T cells, B cells were further reduced, while inflammatory cytokines and D-Dimer continued to increase in severe type patients (Figure 1A). To enhance the immune function of patients and inhibit the formation of inflammatory factor storms, we proposed the following two therapeutic measures.

COVID-19 does not have specific antiviral drug treatment currently, so the treatment of the disease is mainly focused on symptomatic treatment and oxygen therapy. Inflammatory factors and lymphocyte subsets are recommended to be monitored during the disease. We suggest that IVIg and low molecular weight heparin (LMWH) anticoagulant therapy could be given as early as possible when T cells, B cells, inflammatory cytokines, and D-Dimer show the following trends: T lymphocytes and B lymphocytes in peripheral blood are significantly lower than before; inflammatory cytokines such as IL-6 are increased significantly; coagulation parameters such as D-Dimer increased abnormally; Chest CT indicates the expansion of lung lesions. In our recommendation, high-dose IVIg at 0.3–0.5 g per kg weight per day could be given for 5 days, which can interrupt the storm of inflammatory factors at an early stage, enhance immune function. A randomized controlled clinical trial of IVIg in patients with severe SARS-CoV-2 infection has been initiated (NCT 04261426). Although IVIG has shown efficacy in the treatment of patients with influenza [42] and SARS [43], we need more clinical data of COVID-19 patients as evidence.

**Figure 2.**
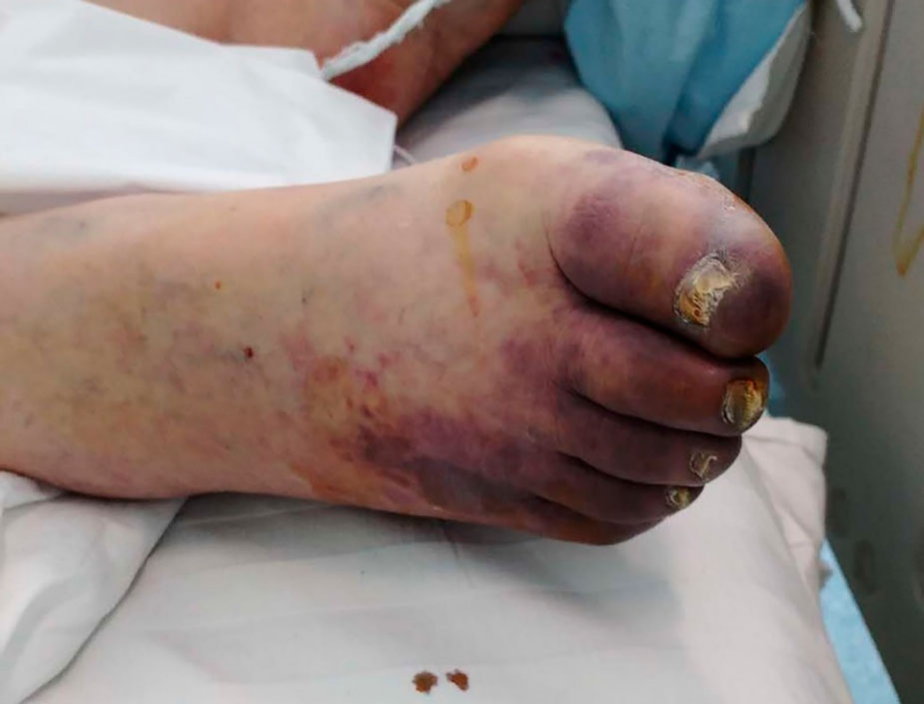
Ischemic changes of toes in one COVID-19 patient with severe type.

LMWH anticoagulation therapy is especially recommended in the early stage of the disease. Infection is a common cause of disseminated intravascular coagulation. Inflammation, infection and other factors can lead to excessive activation of coagulation. We have observed in clinical that COVID-19 patients with severe type may develop disseminated intravascular coagulation (DIC) (unpublished data). In COVID-19 patients with severe type, ischemic changes may occur in the fingers and toes (Figure 2). Anticoagulation therapy is recommended for COVID-19 patients when the D-Dimer value is 4 times higher than the normal upper limit, except for patients with anticoagulant contraindications. The recommended dose of LMWH is 100U per kg weight per 12 h by subcutaneous injection for atleast 3–5 days. Clinicians should closely monitor the indicators of laboratory examination of patients to be alert for side effects after anticoagulant treatment.

In conclusion, the current treatment of COVID-19 patients with severe type and critical type is the key to controlling the rising number of deaths. We recommend early initiation of IVIg and LMWH anticoagulant therapy, which is effective in improving the prognosis of severe and critical type patients. Figure 1B describes the optimal time to initiate IVIg and LMWH anticoagulant therapy, as well as the possible trend of T cells, B cells, Inflammatory cytokines, D-Dimer after therapy. More immune-related research is needed to help us understand the pathogenesis, guide the treatment of the disease, and improve the prognosis.
